# Lip and tongue strength associated with chewing patterns in aging population

**DOI:** 10.1186/s12903-023-03503-z

**Published:** 2023-11-11

**Authors:** Yi-Fang Huang, Wei-Han Chang, Yu-Fang Liao, Mei-Hui Chen, Chung-Ta Chang

**Affiliations:** 1 Department of General Dentistry, Chang Gung Memorial Hospital, Linkou, 33305 Taiwan; 2 School of Dentistry, College of Oral Medicine, Taipei Medical University, Taipei, 11031 Taiwan; 3 Graduate Institute of Dental and Craniofacial Science, College of Medicine, Chang Gung University, Taoyuan, 33302 Taiwan; 4 Department of Physical Medicine and Rehabilitation, Keelung Chang Gung Memorial Hospital, No. 222, Maijin Rd., Anle Dist, Keelung City, 204201 Taiwan; 5 School of Traditional Chinese Medicine, Chang Gung University, Taoyuan, 33302 Taiwan; 6 Department of Craniofacial Orthodontics, Chang Gung Memorial Hospital, Taipei, 10507 Taiwan; 7 Craniofacial Center, Chang Gung Memorial Hospital, Taoyuan, 33378 Taiwan; 8 Craniofacial Research Center, Chang Gung Memorial Hospital, Linkou, 33305 Taiwan; 9 College of Medicine, Chang Gung University, Taoyuan, 33302 Taiwan; 10 Department of Physical Medicine and Rehabilitation, Chang Gung Memorial Hospital, Linkou, 33305 Taiwan; 11 Department of Emergency Medicine, Far Eastern Memorial Hospital, No. 21, Sec. 2, Nanya S. Rd., Banciao Dist, New Taipei City, 22056 Taiwan; 12 Graduate Institute of Medicine, Yuan Ze University, Taoyuan, 32003 Taiwan

**Keywords:** Tongue strength, Lip strength, chewing time, Chewing strokes, Chewing ability, Older adults

## Abstract

**Background:**

Improving chewing function of older adults increases the health-related quality of life. Few studies indicated the correlation between tongue, lip strength on masticatory performance in older people. The study aimed to investigate the association between lip, tongue strength on chewing pattern in aging population.

**Methods:**

The older adults had independent daily intake without assistance were enrolled. They had intact dentition and no periodontitis. To estimate the number of chewing strokes and chewing time by consuming a cornstarch cookie were used to represent chewing pattern. Lip and tongue pressure were evaluated with an Iowa Oral Performance Instrument. Linear regression analysis was used to analyze the lip and tongue pressure associated with the chewing time and strokes. Spearman’s correlation analysis was utilized to evaluate the associations among chewing time and chewing strokes or lip and tongue pressure.

**Results:**

35 women and 35 men with an average age of 73.2 years were investigated. Tongue pressure was significantly related to the chewing time and the number of chewing strokes (*p* = 0.01 and 0.03). There was a close association between chewing time and the number of chewing strokes (*p* < 0.0001). The correlation between lip and tongue pressure was significant (*p* < 0.0001).

**Conclusion:**

The tongue strength significantly related to chewing ability in aging population. Increasing the tongue strength greatly reduced the number of chewing strokes and chewing time. Good masticatory ability could increase the motor function of tongue; raising the tongue strength might be able to improve mastication in older adults.

## Background

Mastication is closely related to stomatognathic, neuromuscular and digestive activities [1]. Poor chewing ability leads malnutrition [2] and is also significantly correlated with cognitive impairment or dementia in the older adults [3]. Good masticatory performance is an important factor influencing health-related quality of life (HRQoL) in the aging population [4]. High life expectancy and a low birth rate have led to an increased proportion of older people. Improving the chewing function of older individuals to reduce medical expenses and increase the HRQoL is an important issue in an aging society. The motor functions of the tongue and lips deteriorate with age then affect masticatory performance [5]. Most investigations focus on the dental status regarding chewing ability [6, 7]. The issue of decreased oral physiology with age related to chewing is often neglected. Many previous studies often focused on the perioral pressure related to tooth position and the effect of orthodontic management [8, 9]. Few investigations revealed the impact of tongue and lip functions on masticatory performance, but this issue is crucial for older individuals.

This study focused on the association between lip, tongue strength related masticatory performance in the older adults. The aim of this study was to estimate the association between lip and tongue pressure and habitual chewing patterns, including chewing time, strokes in aging population. The null hypothesis of this study was the close relationship between these oral physiology and chewing function.

## Methods

### Subjects

The healthy older individuals over 65 years old who had independent daily intake without assistance or swallowing problems were enrolled in this study. These subjects were selected from the Dental Clinic of Chang Gung Memorial Hospital from 2020 to 2022. To eliminate the confounders derived from periodontal disease, occluding pair and neuromuscular disorder, all the subjects must strictly exclude these conditions to increase the realism of the study. All the older individuals had no missing teeth and periodontitis. Those who had neuromuscular disorders, dysphagia, temporomandibular disorders, psychiatric problems or received radiotherapy or head and neck surgery were all excluded. All subjects had good ability to follow the study instructions and clearly understand the purpose of this study. Based on our previous investigation [6], all participants were restricted to without any missing teeth or periodontitis to eliminate the impact of dental status on chewing ability. All the subjects receive regular dental follow-up every six months. Their periodontal condition was healthy and kept good maintenance. The plaque index of subjects was restricted in 0–1 and their attachment level was healthy as the base of gingival sulcus is no more than 1 mm apical to the cemento-enamel junction [10]. This study was approved by the Ethical Committee of Chang Gun Memorial Hospital (201801918B0A3). The subjects were enrolled in this study after they had signed an information consent form. These subjects completed the examination of this study at the dental clinic of Chang Gung Memorial Hospital.

### Lip and tongue pressure measurement

**Fig. 1:**
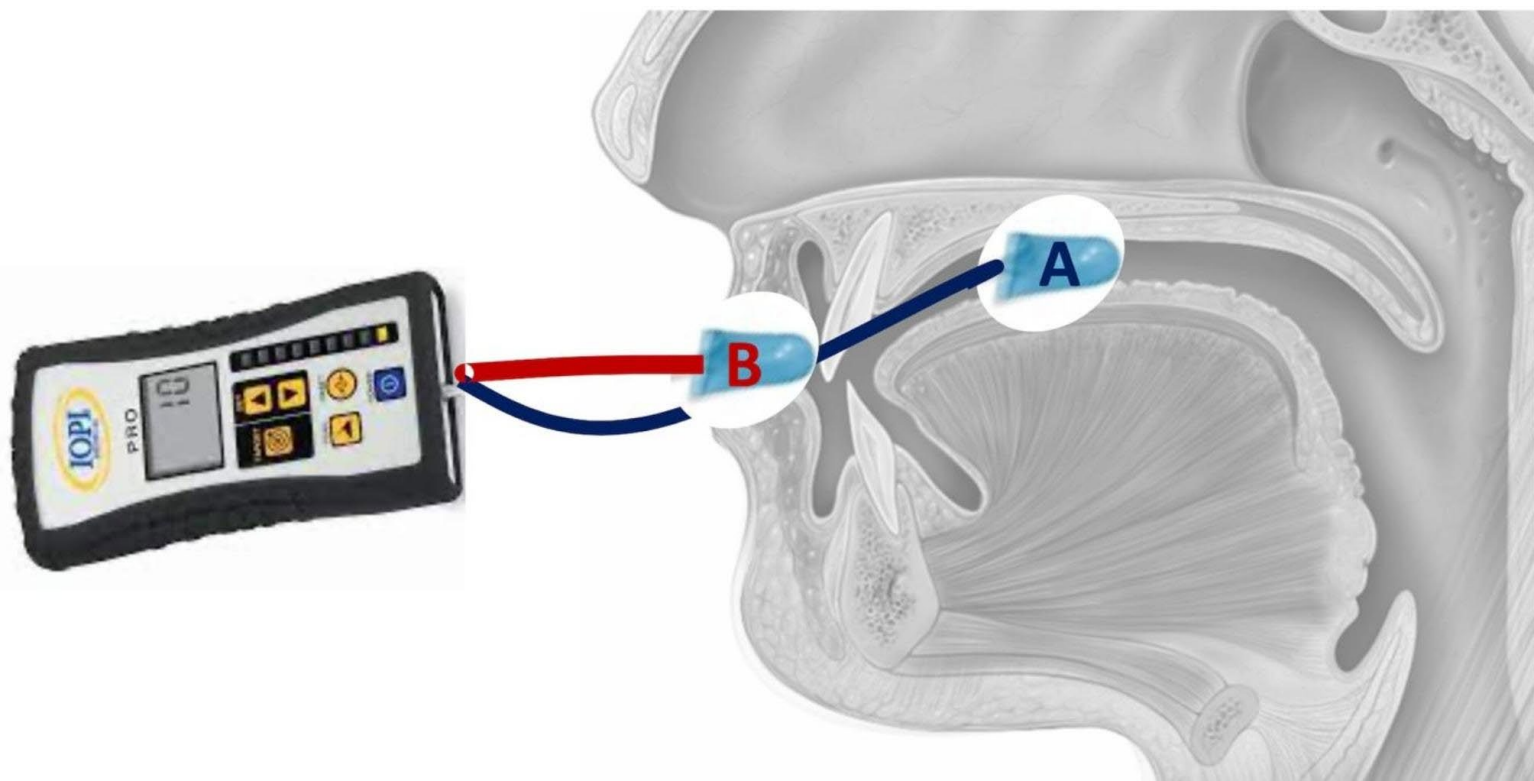
The Iowa Oral Performance Instrument (IOPI) was utilized to measure lip and tongue pressure. The subject was requested to squeeze the bulb (**A**) which placed between tongue and hard palate to evaluate the tongue pressure. The participant was asked to squeeze the bulb (**B**) placed between upper and lower lip to estimate the lip pressure

The Iowa Oral Performance Instrument (IOPI) is a reliable device in lip and tongue pressure evaluation [11]. It measures the pressure generated by compress between the tongue and palate or the upper and lower lip to assess the tongue and lip strength and endurance [12]. The IOPI uses an internal volume 2.8 ml air-filled pliable plastic bulb connected via an 11.5-cm-long clear plastic tube to determine the peak pressure [in kilopascals (kPa)]. It has a peak-hold function, pressure-sensing circuitry and a timer. In this study, the bulb was placed between the tongue and hard palate or upper and lower lip, and then the participant was instructed to squeeze the bulb as hard as possible (Fig. 1). The displayed lip and tongue pressure (kPa) was recorded.

### Chewing time and chewing strokes

To evaluate the amount of chewing strokes and the time required to complete the mastication, all participants were requested to perform their habitual mastication of a cornstarch cookie [6, 13]. The chewing strokes were counted as the opening and closing mandibular movements until tooth contact occurred [14]. The time needed to consume each piece of food was recorded as the chewing time, which was measured with a digital chronometer (Intermec, Everett®, Washington, USA). The chronometer was initialized after placing the food in the oral cavity and ended when each portion was swallowed. This reliable evaluation method was utilized to estimate the chewing pattern of older adults in our previous investigation [6].

### Statistical analysis

The data are presented as the mean and standard deviation (SD). A linear regression analysis was used to analyze the lip and tongue pressure related to the chewing time and strokes. The association between chewing time and chewing strokes or lip pressure and tongue pressure was evaluated with Spearman’s correlation analysis. SAS software version 9.4 (SAS Institute, Cary, NC) was used for these analyses, and a two-tailed test *p* < 0.05 was considered to be statistical significance.

## Results

A total of 70 healthy older adults, including 35 women and 35 men, were enrolled in this study. The age of the subjects ranged from 65 to 74 years (average, 73.2 years). The chewing time was 23.30 ± 23.70 s, the number of chewing strokes was 31.43 ± 34.49, the tongue pressure was 32.69 ± 12.24 KPa, and the lip pressure was 22.39 ± 6.94 Kpa (Table 1). Tongue pressure significantly related to chewing time and chewing strokes (*p* = 0.01 and 0.03, respectively) (Table 2). There was a significant association between the chewing time and chewing strokes (*p* < 0.0001); the lip pressure was closely correlated to tongue pressure (*p* < 0.0001) (Table 3). Hence, this result successfully confirmed the hypothesis of this study.

**Table 1:** The distribution of variables (*N* = 70)

Variable	Mean	SD	Median	Minimum, Maximum
Chewing time	23.30	23.70	18.58	(6.23, 202.20)
Chewing strokes	31.43	34.49	26.00	(10.00, 300.00)
Tongue pressure	32.69	12.24	32.67	(8.67, 57.67)
Lip pressure	22.39	6.94	21.00	(9.17, 46.67)

Abbreviation: *N*, numbers of subjects; SD, standard deviation

The unit of variable: Chewing time = seconds; Chewing strokes = number of times; Tongue and lip pressure = KPa.

**Table 2:** The impact of tongue and lip pressure on chewing time and strokes (*N* = 70)

Outcome	Variable	Estimate	SE	*p*-value
Chewing time	Tongue pressure	-0.57	0.22	0.01*
	Lip pressure	-0.31	0.41	0.46
Chewing strokes	Tongue pressure	-0.75	0.33	0.03*
	Lip pressure	-0.13	0.61	0.83

Abbreviation: *N*, number of subjects; SE, standard error

*p < 0.05

**Table 3:** The correlation between chewing time and chewing strokes

Variable	Variable	R	*p*-value
Chewing strokes	Chewing time	0.98	< 0.0001***
Lip pressure	Tongue pressure	0.58	< 0.0001***

***p < 0.005

## Discussion

This study utilizing tongue and lip pressure to represent the tongue and lip strength was reliably [1, 15]. As previous study proposed the pulverization of food needs more intensive chewing, the complaints related to masticatory function were proved to be consistent with chewing longer [6, 16]. To count the chewing strokes is used to quantitatively evaluate chewing ability [6, 17]. Hence, chewing time and strokes can effectively characterize the masticatory performance. This study preliminarily revealed that there was a close association between tongue strength and chewing ability in the older adults (Table 2). This result is similar to the previous investigation proposed that masticatory function relied on a normal pattern of tongue-to-hard palate contact, the control of tongue activity, and coordination with jaw movement [18]. Based on this result, increasing the tongue strength significantly shortened the chewing time and reduced the number of chewing strokes; good chewing ability might be able to greatly raise the motor function of tongue.

In general, dental status is closely related to the ability to crush food, but less information has been mentioned about the effect of perioral muscle strength on chewing function in the aging population. This result indicated the chewing ability in the older adults was not only correlated to the dental status but also associated with the tongue strength. Increasing tongue motor activity can also actually promote HRQoL because good mastication will increase nutritional intake among older individuals. Diet for the elderly often advocates how to soften food for adapting to the deterioration of chewing ability. Based on this study, the masticatory training might be able to raise the tongue activity in older people to be regarded as a new-style rehabilitative treatment modality. For example, we might infer to increase the food texture might be able to improve the tongue strength. These effects need to be confirmed in further study. This discovery possibly will have multiple practical applications in the geriatric field. The previous investigation proposed a positive correlation between mastication and cognitive function, such as dementia, in older individuals [19]. Based on this result, we could infer tongue activity training to improve masticatory function might alleviate the deterioration associated with age and prevent cognitive disorders. The correlation between tongue strength and cognitive function is worthy to further discussion.

This study revealed the lip strength might be not significantly associated with the chewing ability (Table 2), although the previous study proposed that lip strength is highly correlated with sucking functions during the capture of adhesive food [20]. The variance might be attributed to the different texture of the test food. The subjects of this study were seriously restricted to healthy older adults and those who could eat independently but the most older individuals had multiple systemic diseases. In the future, it will be interesting to investigate the impact of lip strength on nutritional intake in the older adults needing passive feeding because that type of feeding often relies on spoon suction. This discovery indicated the close association between lip and tongue pressure (*p* < 0.0001) (Table 3), so the lip and tongue significantly interacted with each other. Other research revealed that lip strength is significantly related to swallowing dysfunction [21]. According to the result just mentioned, whether chewing training might increase lip motor indirectly through raising the tongue motor activity then prevent or improve dysphagia. This inference needs to be further investigated.

This result revealed the significant correlation between the oral physiology including prei-oral pressure and chewing patterns. Based on this investigation, future research will focus on finding linear relationships between various oral physiological conditions under large aging population. That might be able to provide valuable clinical significance to link the clinical evaluation related to the oral function. Although this study had multiple strengths, there were some limitations. Tooth loss is the most common predicament in older individuals and greatly impacts mastication function and chewing efficiency [22, 23]. Posterior occlusal support is closely related to masticatory performance [6]. To clarify the impact of lip and tongue strength on mastication, all the participants were restricted to full intact dentition and have no periodontitis. Although this restriction of participant increases the realism, those who cannot represent all aging population. Because the periodontal disease is the most common disease to lead missing teeth in older adults, further study should consider the impact of remaining dentition on lip and tongue strength. In the future, it would be interesting to investigate the possibility of tongue strength or masticatory function according to this results with potential occlusal deteriorations or abfractions whether through different occlusal load and frequency of eccentric dynamic occlusion.

## Conclusions

There was a close association between tongue strength and chewing ability in the older adults. In the aging population, increasing the tongue strength significantly decreased the chewing strokes and shortened the chewing time. Improving chewing ability might be able to raise the motor function of tongue.

## Data Availability

The data sets generated and/or analyzed during the present study are available from the corresponding author on reasonable request.

## Abbreviations

HRQoL: health-related quality of life
IOPI: Iowa Oral Performance Instrument
SD: standard deviation

## References

[1] Ramos VF, Silva AF, Degan VV, Celeste LC, Picinato-Pirola M (2023). Lip and tongue pressure and the functionality of oro-facial structures in healthy individuals. J Rehabil.

[2] Cin P, Tanrıöver Ö, Yavuzer H, Erdinçler DS (2021). Evaluation of Malnutrition status and related risk factors in geriatric outpatient clinic. Nutr Res Pract.

[3] Kim EK, Lee SK, Choi YH, Tanaka M, Hirotsu K, Kim HC (2017). Relationship between chewing ability and cognitive impairment in the rural elderly. Arch Gerontol Geriatr.

[4] Cho MJ, Kim EK (2019). Subjective chewing ability and health-related quality of life among the elderly. Gerodontology.

[5] Kato Y, Kikutani T, Sagawa K, Tamura F (2022). Association between masticatory movement and oral and physical function in healthy older women. J Prosthodont Res.

[6] Huang Y-F, Liu S-P, Muo C-H, Chang C-T (2021). The impact of occluding pairs on the chewing patterns among the elderly. J Dent.

[7] Medeiros MMD, Figueredo OMC, Pinheiro MA, Oliveira LFS, Wanderley RL, Araújo ECF (2022). Prosthetic rehabilitation status, dental prosthesis functionality and masticatory function in nursing home residents. Gerodontology.

[8] Lambrechts H, De Baets E, Fieuws S, Willems G (2010). Lip and tongue pressure in orthodontic patients. Eur J Orthod.

[9] Koletsi D, Makou M, Pandis N (2018). Effect of orthodontic management and orofacial muscle training protocols on the correction of myofunctional and myoskeletal problems in developing dentition. A systematic review and meta-analysis. Orthod Craniofac Res.

[10] Karmakar S, Prakash S. Clinical attachment level: an unsung hero in periodontal diagnosis. Int J Adv Res. 2019.

[11] Martins FAG, Motta AR, Neves LS, Furlan R (2023). Evaluation of the maximum tongue and lip pressure in individuals with class I, II, or III Angle malocclusions and different facial types. Codas.

[12] IOPI Medical LLC. Iowa Oral Performance Instrument: users manual. 2020.

[13] Picinato-Pirola MN, Mello-Filho FV, Trawitzki LV (2012). Chewing time and chewing strokes in different dentofacial deformities. J Soc Bras Fonoaudiol.

[14] Fassicollo CE, Garcia DM, Machado BCZ, de Felício CM (2021). Changes in jaw and neck muscle coactivation and coordination in patients with chronic painful TMD disk displacement with reduction during chewing. Physiol Behav.

[15] Ryu M, Oki T, Ohta M, Sakurai K, Ueda T (2021). Effect of lip-seal resistance training on lip-seal strength in young adults. Bull Tokyo Dent Coll.

[16] Park S, Kim DK, Park H, Yoon D, Byambaa S (2022). Improvement of chewing and swallowing risks in community-dwelling older adults using texture-modified food. Nutr Res Pract.

[17] Nagaiwa M, Gunjigake K, Yamaguchi K (2016). The effect of mouth breathing on chewing efficiency. Angle Orthod.

[18] Park YS, Hong HP, Ryu SR, Lee S, Shin WS (2022). Effects of textured food masticatory performance in older people with different dental conditions. BMC Geriatr.

[19] Tada A, Miura H (2017). Association between mastication and cognitive status: a systematic review. Arch Gerontol Geriatr.

[20] Nakamura Y, Nakajima T, Sasakawa Y, Tsukuno S, Sakurai R, Kurosawa M (2020). Influence of food adhesivity and quantity in lip closing pressure. Physiol Behav.

[21] Messina G, Giustino V, Martines F, Rizzo S, Pirino A, Scoppa F (2019). Orofacial muscles activity in children with swallowing dysfunction and removable functional appliances. Eur J Transl Myol.

[22] Hoeksema AR, Peters LL, Raghoebar GM, Meijer HJA, Vissink A, Visser A (2017). Oral health status and need for oral care of care-dependent indwelling elderly: from admission to death. Clin Oral Investig.

[23] Kumar Y, Chand P, Arora V, Singh SV, Mishra N, Alvi HA, Verma UP (2017). Comparison of rehabilitating missing Mandibular First molars with Implant- or tooth-supported prostheses using Masticatory efficiency and patient satisfaction outcomes. J Prosthodont.

